# Differential Functional Connectivity Alterations of Two Subdivisions within the Right dlPFC in Parkinson's Disease

**DOI:** 10.3389/fnhum.2017.00288

**Published:** 2017-05-30

**Authors:** Julian Caspers, Christian Mathys, Felix Hoffstaedter, Martin Südmeyer, Edna C. Cieslik, Christian Rubbert, Christian J. Hartmann, Claudia R. Eickhoff, Kathrin Reetz, Christian Grefkes, Jochen Michely, Bernd Turowski, Alfons Schnitzler, Simon B. Eickhoff

**Affiliations:** ^1^Department of Diagnostic and Interventional Radiology, Medical Faculty, University DüsseldorfDüsseldorf, Germany; ^2^Research Centre Jülich, Institute of Neuroscience and Medicine (INM-1, INM-3, INM-11)Jülich, Germany; ^3^Medical Faculty, Institute of Clinical Neuroscience and Medical Psychology, Heinrich-Heine-UniversityDüsseldorf, Germany; ^4^Department of Neurology, Medical Faculty, Center for Movement Disorders and Neuromodulation, Heinrich-Heine-UniversityDüsseldorf, Germany; ^5^Department of Psychiatry, Psychotherapy and Psychosomatics, RWTH Aachen UniversityAachen, Germany; ^6^JARA BRAIN and Department of Neurology, RWTH Aachen UniversityAachen, Germany; ^7^Department of Neurology, University of CologneCologne, Germany

**Keywords:** executive function, cognitive motor control, working memory, resting-state fMRI, levodopa

## Abstract

Patients suffering from Parkinson's disease (PD) often show impairments in executive function (EF) like decision-making and action control. The right dorsolateral prefrontal cortex (dlPFC) has been strongly implicated in EF in healthy subjects and has repeatedly been reported to show alterations related to EF impairment in PD. Recently, two key regions for cognitive action control have been identified within the right dlPFC by co-activation based parcellation. While the posterior region is engaged in rather basal EF like stimulus integration and working memory, the anterior region has a more abstract, supervisory function. To investigate whether these functionally distinct subdivisions of right dlPFC are differentially affected in PD, we analyzed resting-state functional connectivity (FC) in 39 PD patients and 44 age- and gender-matched healthy controls. Patients were examined both after at least 12 h withdrawal of dopaminergic drugs (OFF) and under their regular dopaminergic medication (ON). We found that only the posterior right dlPFC subdivision shows FC alterations in PD, while the anterior part remains unaffected. PD-related decreased FC with posterior right dlPFC was found in the bilateral medial posterior parietal cortex (mPPC) and left dorsal premotor region (PMd) in the OFF state. In the medical ON, FC with left PMd normalized, while decoupling with bilateral mPPC remained. Furthermore, we observed increased FC between posterior right dlPFC and the bilateral dorsomedial prefrontal cortex (dmPFC) in PD in the ON state. Our findings point to differential disturbances of right dlPFC connectivity in PD, which relate to its hierarchical organization of EF processing by stronger affecting the functionally basal posterior aspect than the hierarchically higher anterior part.

## Introduction

Although Parkinson's disease (PD) is predominantly determined by motor symptoms, cognitive deficits are increasingly recognized as key features of the disorder, having profound impact on patient's quality of life (Michely et al., 2012; Santos-Garcia and de la Fuente-Fernandez, 2013; Berganzo et al., 2014). Affection of cognitive abilities in PD has mainly been attributed to disturbance of frontostriatal circuits caused by dopaminergic depletion (Owen, 2004; Leh et al., 2010). In this regard, the prefrontal cortex, and the dorsolateral prefrontal cortex (dlPFC) in particular, have been repeatedly reported by neuroimaging studies to show alterations in their function and connectivity in PD (e.g., Cools et al., 2002; Lewis et al., 2003; Monchi et al., 2007; Hirano et al., 2012; Disbrow et al., 2013). The observed alterations in dlPFC function have been mainly associated with PD related impairment in executive function (EF). EF describes abilities to handle simultaneous operations, decision-making, pursue personal goals, and cope with novel situations (Kudlicka et al., 2011; Baddeley, 2012; Lezak et al., 2012). It is closely linked to working memory, selective attention and inhibitory processes (Alvarez and Emory, 2006; Muller et al., 2015). In PD, a broad spectrum of EF components have been found to be impaired throughout all stages of the disease comprising deficits in attention and internal action control, set shifting, planning, and conflict resolution, to mention a few (Dirnberger and Jahanshahi, 2013). Impaired performance of PD patients compared to healthy controls in several neuropsychological EF tests like the Wisconsin Card Sorting Test (WCST), the Stroop task, Trail Making Test, tower tests (e.g., Tower of London), or verbal fluency tasks was repeatedly reported (Kudlicka et al., 2011). Furthermore, it could be demonstrated that deficits in EF tests are a predictor for the development of dementia in PD (Levy et al., 2002; Janvin et al., 2005).

The prefrontal cortex is a key structure for EF in the human brain (Miller and Cohen, 2001). Particularly, the dorsolateral prefrontal cortex (dlPFC) is thought to realize EF through top-down modulation of behavior in connection with diverse cortical and subcortical regions (MacDonald et al., 2000; Petrides, 2005; Mansouri et al., 2009). For this purpose, the dlPFC monitors ongoing actions in alignment to internal goals, integrates information from sensory systems and adjusts working-memory information for goal-directed behavior (Ridderinkhof et al., 2004a). In this regard, the dlPFC is functionally organized in a posterior-to-anterior directed hierarchy, where posterior parts process more basal aspects of EF like stimulus integration, while the anterior parts implement more abstract and supervisory functions (Koechlin et al., 2003; Badre and D'Esposito, 2009). Although, the dlPFC was revealed by several studies as a target of altered functional connections in PD (e.g., Wu et al., 2012; Wen et al., 2013; Szewczyk-Krolikowski et al., 2014; Amboni et al., 2015; Trujillo et al., 2015), there is yet no systematic investigation specifically evaluating the disease related functional connectivity (FC) alterations of the dlPFC in PD with respect to its functional and hierarchical sub-regions.

**Figure 1:**
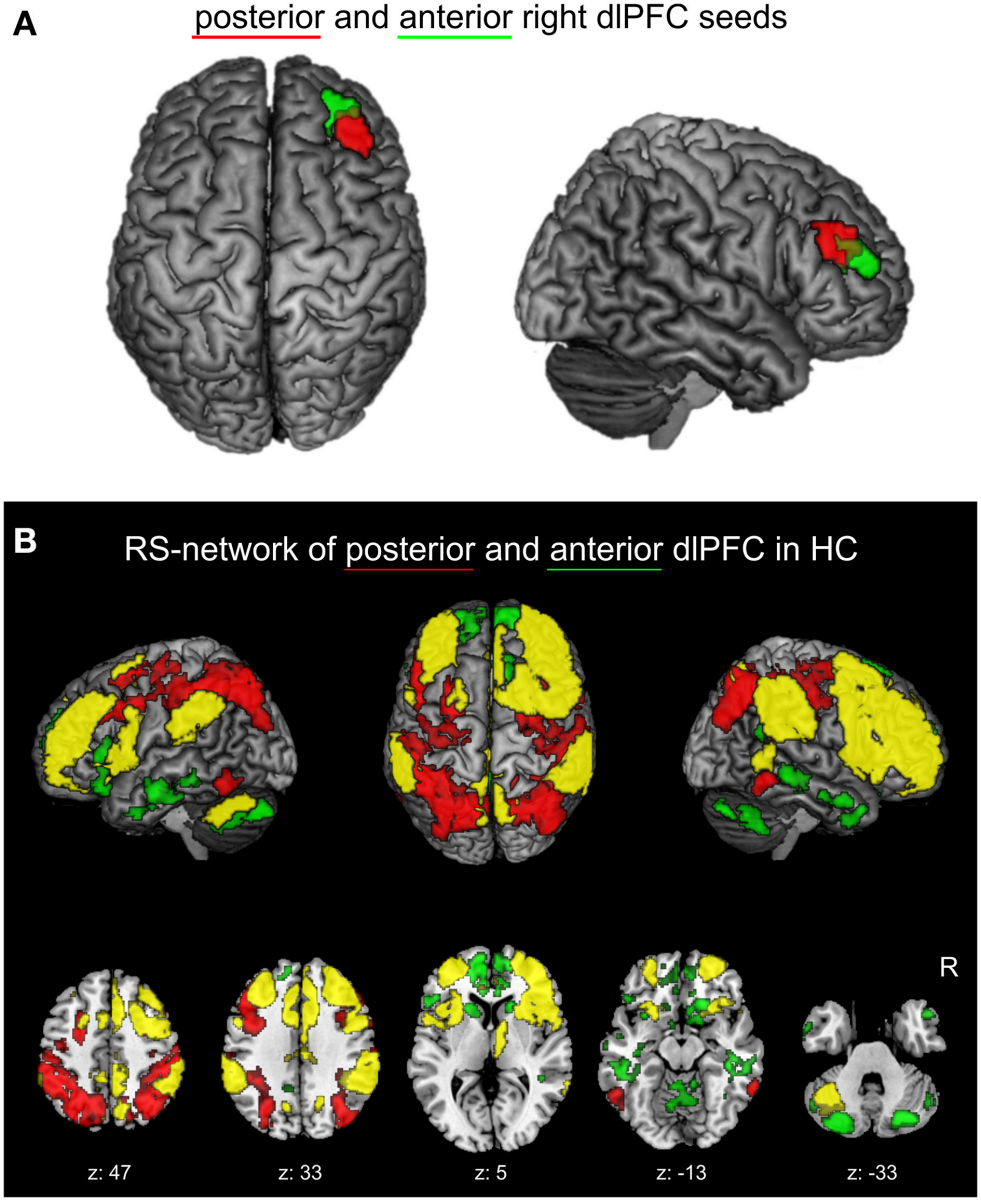
**(A)** Right dlPFC seed regions. Posterior (red) and anterior (green) right dlPFC seeds derived from previous co-activation based parcellation (Cieslik et al., 2013) are projected onto the MNI single subject brain. Top and right lateral views are shown. **(B)** Positively correlated functional connectivity (FC) network of the two right dlPFC seed regions in healthy controls. The conjunction (yellow) of the anterior and posterior right dlPFC seeds as well as the contrasts between the FC networks of both seeds (red: posterior > anterior; green: anterior > posterior) are shown. Regions with significant functional connectivity (cluster-level family wise error corrected, *p* < 0.05; cluster-forming threshold at voxel-level: *p* < 0.001) with the seeds are projected onto the MNI single subject brain. Left lateral, top and right lateral views (top row) as well as five representative sections (bottom row) are shown. Labels under axial sections represent z-coordinates in MNI space. R: right side.

Recently, a functional partitioning within the right dlPFC was revealed by co-activation based parcellation (Cieslik et al., 2013). In this study, a region at the inferior frontal sulcus involved in cognitive action control was partitioned into an anterior and posterior subdivision based on their whole-brain co-activation profiles (Figure 1A). Characterization of the functional involvement and connectivity of the two sub-regions confirmed the posterior-to-anterior hierarchical organization for EF. In particular, the posterior subdivision is connected more strongly to posterior parietal areas and is more involved in visuomotor stimulus integration and working memory processes. The anterior right dlPFC sub-region is connected more strongly to the anterior cingulate cortex (ACC) and facilitates supervisory functions involving more abstract processes of EF, especially concerning conflict resolution.

In PD, both, basal EF like stimulus integration or working memory updating as well as its more abstract aspects like set-shifting or inhibition can be affected (Ranchet et al., 2011; Dirnberger and Jahanshahi, 2013). With respect to the diversity of EF impairment in PD, the question arises, if the two functionally distinct right dlPFC subregions are differentially affected in PD. Thus, the aim of the current study was to investigate, if the connectivity patterns of the anterior and posterior right dlPFC clusters are differentially altered in PD patients, in order to achieve deeper insights in the pathophysiology underlying EF impairment in PD. To this end, we investigated resting-state functional connectivity (FC) of a sample of PD patients compared to a group of matched healthy controls, seeding from the anterior and posterior right dlPFC sub-regions described above (Cieslik et al., 2013). In order to test for dopaminergic effects in PD, we investigated FC of PD patients both after at least 12 h withdrawal of dopaminergic drugs (medical OFF) and under their regular medication (medical ON). Moreover, we assessed the relationship between changes in FC and disease-related clinical parameters, such as the severity of cognitive and motor symptoms of PD patients.

## Materials and methods

### Sample

Thirty-nine patients (12 females) from the University Hospital Düsseldorf diagnosed with idiopathic Parkinson's disease (iPD) with a mean age of 62.2 years (range 44–80) and 44 healthy volunteers (21 females) with a mean age of 59.4 years (range 41–81) and without any record of neurological or psychiatric disorder were included in the current analysis (see Table 1). All subjects provided written informed consent to participate in the study prior to inclusion. The study was approved by the local ethics committee of the Heinrich-Heine-University Düsseldorf Medical Faculty.

**Table 1:** Age, gender, and within-scanner movement parameters of the study sample.

**Group**	***n***	**Age: mean (±sd)**	**P (*t*-test)**	**Gender: females (%)**	**P (χ^2^- test)**	**RMS: mean (±sd)**	**P (*t*-test)**	**DVARS: mean (±sd)**	**P (*t*-test)**	**FD: mean (±sd)**	**P (*t*-test)**
Patients	39	62.6 (±9.1)	0.119	12 (31%)	0.115	0.36 (±0.16)	0.226	1.93 (±0.51)	0.140	0.51 (±0.22)	0.115
Controls	44	59.4 (±9.6)		21(47%)		0.31 (±0.24)		1.77 (±0.51)		0.41 (±0.31)	

*Movement parameters refer to patient's scans under their regular dopaminergic medication (medical ON). RMS, root mean squared movement; DVARS, root mean squared signal change across time-series; FD, framewise displacement*.

Patients and healthy controls were matched for age, gender, and importantly also for within-scanner head movement. For this, we determined the largest sample pool, in which patient's age, gender, and estimates of head movement derived from EPI motion correction were not significantly different from those of healthy controls. These motion parameters used for movement matching were root mean squared movement (RMS), root mean squared signal change across time-series (DVARS), and framewise displacement (FD) (Power et al., 2012). Matching was accomplished by iteratively drawing random samples for each group and performing two-sample *t-*tests for each parameter between the groups (10^7^ iterations, *p* > 0.1). Movement matching was performed using the scans of PD patients when under their regular dopaminergic medication (medical ON). This is the more conservative approach compared to using scans OFF medication in regard to movement matching, as patients generally show less global bradykinesia when ON dopaminergic treatment.

For each patient, diagnosis of iPD was made by the attending neurologist at the Department of Neurology, University Hospital Düsseldorf according to the International Classification of Diseases (ICD-10). Exclusion criteria were severe dementia, major depression or any psychological or medical condition that could interfere with the conduction of the study. All patients were treated with an individual PD-related medication scheme resulting from optimization of treatment by the attending neurologist, including levodopa (for all patients), catechol-O-methyl-transferase (COMT) inhibitors, dopamine agonists as well as further symptomatic drugs. To make these treatment regiments consistent on a unified scale, we calculated the levodopa equivalent dose (LED) according to Tomlinson et al. (2010).

FMRI scans as well as clinical measures were assessed while PD patients were on their regular medication (medical ON). Furthermore, an additional fMRI scan was conducted after overnight withdrawal of the dopaminergic medication for at least 12 h (medical OFF). PD-related motor symptoms were quantified by the Unified Parkinson's Disease Rating Scale part III (UPDRS-III) in the medical ON as well as the medical OFF by the attending neurologist in close proximity to fMRI scans. Dopaminergic medication induced a significant improvement of the UPDRS-III motor score in patients (OFF mean = 35.2; ON mean = 21.1; *p* < 0.001), indicating that patients showed a strong response to their regular medication. Additionally, Mattis dementia rating scale (MDRS) scores were assessed for all patients and Montreal Cognitive Assessment (MoCA) scores were assessed for 33 of 39 patients in the medical ON. The characteristics of the patient sample regarding disease duration, Hoehn and Yahr stage, UPDRS-III scores, MDRS score, MoCA score, LED, motor subtypes and lateralization of symptoms, are given in Table 2 and Supplementary Table S1.

**Table 2:** PD-related characteristics and measures of the patient sample.

**Disease duration (years)**	**Hoehn and Yahr**	**UPDRS-III (medical OFF)**	**UPDRS-III (medical ON)**	**MDRS (medical ON)**	MoCA^*^ (medical ON)	**LED [mg]**	**Motor subtype (AR | TD | MT)**	**Symptom lateralization (right | left)**
8.9 (±5.7) [0–21]	2.6 (±0.7) [1–4]	35.2 (±11.5) [15–57]	21.1 (±10.9) [6–47]	137.1 (±6.4) [118–144]	24.5 (±3.7) [16–29]	1023.0 (±435.0) [100–1,900]	13 | 5 | 21 (33% | 13% | 54%)	16 | 23 (41% | 59%)

Values for disease duration, Hoehn and Yahr stage, UPDRS-III, MDRS and LED are mean (±standard deviation) [value range]. UPDRS-III, Unified Parkinson's Disease Rating Scale part III; MDRS, Mattis Dementia Rating Scale. MoCA, Montreal Cognitive Assesment (

* *MoCA was available in 33 of 39 patients). LED, Levodopa equivalent dose; AR, akinetic-rigid; TD, tremor-dominant; MT, mixed type*.

### MRI data acquisition and preprocessing

For each participant, resting-state fMRI was acquired using an echo planar imaging (EPI) sequence of the whole brain (vertex to lower cerebellum) to obtain blood oxygen level dependent (BOLD) time-series. Participants were instructed to lie still in the scanner and to “not think of anything in particular,” but not to fall asleep during the scan. Image acquisition was conducted on a Siemens Trio 3T at the Department of Diagnostic and Interventional Radiology, University Hospital Düsseldorf using the following scanning parameters: TR = 2.2 s, TE = 30 ms, flip angle = 90°, 36 slices with an isotropic voxel size of 3.1 × 3.1 × 3.1 mm^3^. Three hundred EPI images were acquired over an interval of 11 min.

Preprocessing of MRI datasets was conducted with SPM12 (http://www.fil.ion.ucl.ac.uk/spm). To account for magnetic field saturation effects, the first four images of each BOLD time-series were discarded. Within-scanner motion correction was applied using a two-pass affine registration procedure. That is, images were initially realigned to the first image of the dataset and then subsequently to the mean of the realigned images. As part of this procedure, the following parameters were calculated, which were used for movement matching between patients and healthy controls (see above): RMS, DVARS, and framewise displacement (FD) (Power et al., 2012).

For each participant, the resulting mean EPI image was spatially normalized to the MNI152 non-linear template (MNI, Montreal Neurological Institute) using the unified segmentation approach (Ashburner and Friston, 2005). The ensuing deformations were then applied to each individual EPI volume. Image volumes were resampled to a voxel size of 1.5 × 1.5 × 1.5 mm^3^ within the normalization procedure. To improve signal-to-noise ratio, images were spatially smoothed using a 5 mm full-width at half-maximum (FWHM) Gaussian kernel.

In order to reduce spurious correlations evoked by inevitable confounds such as physiological noise and motion (Bandettini and Bullmore, 2008), variance explained by the following nuisance variables was removed from each voxel's time series: (i) the six motion parameters obtained from spatial realignment, (ii) square values of these motion parameters, (iii) the first derivatives of the six motion parameters, (iv) square values of these derivatives, (v) signal intensity of white matter and CSF (Jakobs et al., 2012; Satterthwaite et al., 2013). The application of global signal regression is critically debated, as it is, on the one hand, an effective strategy to control for several confounds, but, on the other hand, can evoke bias to the data (Murphy et al., 2009; Power et al., 2014). As yet, there is still no consensus on what is the most valid approach for nuisance removal. We here used signal intensity of white matter and CSF for nuisance removal instead of a global signal regression. Subsequently, data was band-pass filtered, preserving frequencies between 0.01 and 0.08 Hz (Fox and Raichle, 2007; zu Eulenburg et al., 2012).

### Definition of seed regions

The volumes of interest (VOIs) of the anterior and posterior right dlPFC clusters from a previous co-activation based parcellation study of the right dlPFC (Cieslik et al., 2013) were used as seed regions (Figure 1A). In this previous study, a functionally defined region within the right dlPFC, derived from four different functional magnetic resonance imaging (fMRI) experiments concerning cognitive action control, was separated into two distinct clusters using a hierarchical cluster analysis and spectral reordering on the co-activation matrices of the region's voxels. Co-activation profiles of voxels were based on an activation likelihood estimation (ALE) approach of those experiments in the BrainMap database (Laird et al., 2011; http://www.brainmap.org, Laird et al., 2009) that featured activation foci within the right dlPFC (Eickhoff et al., 2012).

The first eigenvariates of VOI time-series were calculated separately for the anterior and posterior right dlPFC seed and supplied to further whole-brain FC analysis in our sample of PD patients and healthy controls.

### Functional connectivity analysis

Whole-brain FC of the anterior and posterior right dlPFC seeds was analyzed by correlation of each seed's time-series, more precisely their first eigenvariates, with the time-series of all gray-matter voxels. The resulting Pearson correlation coefficients for each voxel were then transformed into Fisher's Z scores, which represented the connectivity in each individual gray-matter voxel with the respective seed. Group statistics on the yielded FC maps across all subjects was carried out by an analysis of variance applying a multivariate general linear model (GLM).

To characterize the physiological networks of both right dlPFC seed regions, we investigated the brain regions showing significant positive FC with the seeds in healthy controls in a GLM. To reveal commonalities and differences between the networks of the anterior and posterior right dlPFC seeds, a conjunction between the positively correlated networks of both seeds, as well as difference maps (anterior > posterior right dlPFC and posterior > anterior right dlPFC) were calculated. Results from these conjunction and contrast analyses were cluster-level family-wise error (FWE) corrected for multiple comparisons at *p* < 0.05 (cluster-forming threshold at voxel-level: *p* < 0.001).

### Group-differences in whole-brain functional connectivity

Group differences between patients and controls were analyzed for both medical conditions (“patient medical OFF vs. healthy control” and “patient medical ON vs. healthy control”) in separate GLM's. Each model comprised subject group (“patient” or “control”) and seed (“anterior” or “posterior” right dlPFC) as grouping variables. For each seed region, we then tested for group differences between patients and controls. In order to restrict resulting connectivity alterations to areas showing FC with the seed, group differences were calculated in conjunction with the positively correlated network of the respective seed region in either healthy controls or patients. To analyze seed-specific FC differences between patients and healthy controls, that is, FC group differences that are significantly higher for one seed compared to the other, we tested for the “seed × subject group” interaction effects in conjunction with the positively correlated network of the respective seed in the respective subject group.

To investigate the dopaminergic effects in PD patients, we additionally performed a FC comparison between the two medical conditions of PD patients (“patient medical OFF vs. patient medical ON”) in a separate GLM with a paired *t-*test design. In this GLM, LED was modeled as a covariate for both medical conditions. Differences between the ON and OFF medical states were then tested for each seed.

To control for multiple comparisons, results from each group comparison were considered significant, if they passed a threshold at a cluster-level family wise error (FWE) rate of *p* < 0.05 within the positively correlated network of each seed (cluster-forming threshold at voxel level: *p* < 0.001).

### *Post-hoc* network FC analysis

In order to investigate the effect of dopaminergic treatment in brain regions showing altered FC between patients and controls more precisely, we performed a *post-hoc* network FC analysis of these regions between the medical ON and the OFF state in all patients. For this, we extracted VOIs of the clusters showing significant group differences in the “patient medical ON vs. healthy control” or the “patient medical OFF vs. healthy control” group comparisons from the whole-brain FC analysis. As the anterior right dlPFC showed no significant FC group differences, VOIs were only extracted for regions with altered FC with the posterior right dlPFC. For each VOI, the first eigenvariate of their voxel's timeseries was calculated and correlated with the timeseries' eigenvariate of the seed. Resulting Pearson correlation coefficients were transformed into Fisher's Z scores and a paired *t-*test was conducted across all patients for each VOI (*p* < 0.05).

### Correlations between functional connectivity and clinical parameters

We investigated possible correlations of the seeds' FC with disease duration, UPDRS-III motor score, MDRS, MoCA, and LED. We performed whole-brain correlation analyses of FC with the seeds as well as targeted correlation analyses of the FC between regions with altered connections from the previous whole-brain group comparisons.

For each covariate, significant whole-brain correlations with the seeds' FC were investigated in separate GLMs, each including the respective covariate as an additional regressor to the group difference GLMs described above. Disease duration and UPDRS-III were tested for both, the medical ON and the medical OFF state, in separate GLMs, where the UPDRS-III score acquired in the respective medical condition (ON or OFF) was used. Additionally, we tested for the “medication state × UPDRS-III” interaction for each seed region using the patient (ON vs. OFF) GLM. As MDRS was assessed in the medical ON, correlation analysis was only performed for this condition. Correlation analysis of LED with FC was conducted for the medical ON state using the patients (medical ON) vs. healthy controls group comparison GLM, as well as for the FC difference between medical ON state and medical OFF state of PD patients using the patient GLM (ON vs. OFF). FC difference between both medical states was also correlated with disease duration. Correlation results were calculated in conjunction with the respective seed's positively correlated network in patients and cluster-level family wise error (FWE) corrected at *p* < 0.05 (cluster-forming threshold at voxel level: *p* < 0.001).

The correlation analyses with the FC of altered connections were performed in a *post-hoc* network analysis using the VOIs of brain regions showing significant group differences in the “patient medical OFF vs. healthy control” or the “patient medical ON vs. healthy control” whole-brain FC group comparisons. For each significant connection from whole-brain group comparisons, Pearson correlations between the FC of the respective connection and each covariate were calculated (*p* < 0.05). Again, correlations with disease duration and UPDRS-III were tested for both, the medical ON and the medical OFF state, where the UPDRS-III score acquired in the respective medical condition (ON or OFF) was used. Correlations with MDRS and MoCA were only performed for the medical ON condition. Correlation analysis of LED with FC was conducted for the medical ON state, as well as for the FC difference between medical ON state and medical OFF state of PD patients.

### Anatomical allocation of results

All brain regions resulting from connectivity analyses were anatomically allocated to probabilistic cytoarchitectonic maps of JuBrain, the Jülich brain Atlas (Zilles and Amunts, 2010) using the SPM Anatomy Toolbox (http://www.fz-juelich.de/ime/spm_anatomy_toolbox, V.2.0 Eickhoff et al., 2005, 2007).

## Results

### Physiological right dlPFC networks

The physiological positively correlated networks of the anterior and posterior right dlPFC seeds in healthy controls are shown in Figure 1B. Both seeds are connected to a broad network of commonly connected brain regions, but also show several distinctions in their FC.

The common network is largely symmetric and comprises the bilateral dlPFC and right ventrolateral prefrontal cortex (vlPFC), the bilateral dorsomedial prefrontal cortex (dmPFC), the anterior, mid- and posterior cingulate cortex, the bilateral premotor cortex, the bilateral medial posterior parietal cortex (mPPC), the bilateral inferior parietal lobule, the anterior insula, the right thalamus, and left cerebellar lobule VII.

Contrast analysis of physiological FC between both seed regions revealed that, compared to the posterior seed, the anterior right dlPFC is stronger connected to the bilateral anterior dmPFC, the bilateral anterior and left posterior cingulate cortex, the medial and inferior temporal lobes, the bilateral caudate nucleus the medial anterior lobe of the cerebellum and cerebellar lobule VII bilaterally.

Regions showing stronger FC with the posterior compared to the anterior right dlPFC are found in the bilateral dlPFC, posterior parietal areas, i.e., the intraparietal sulcus as well as posterior superior and inferior parietal lobule bilaterally, in the dorsal premotor cortex (PMd) bilaterally and the bilateral ventral occipitotemporal cortex.

The observed networks of the anterior and posterior right dlPFC seed regions in the current sample are well in line with the connections of the seeds from our previous study (Cieslik et al., 2013), where resting-state functional connectivity was investigated in a different sample of 100 healthy subjects.

### Group differences: PD vs. HC

**Figure 2:**
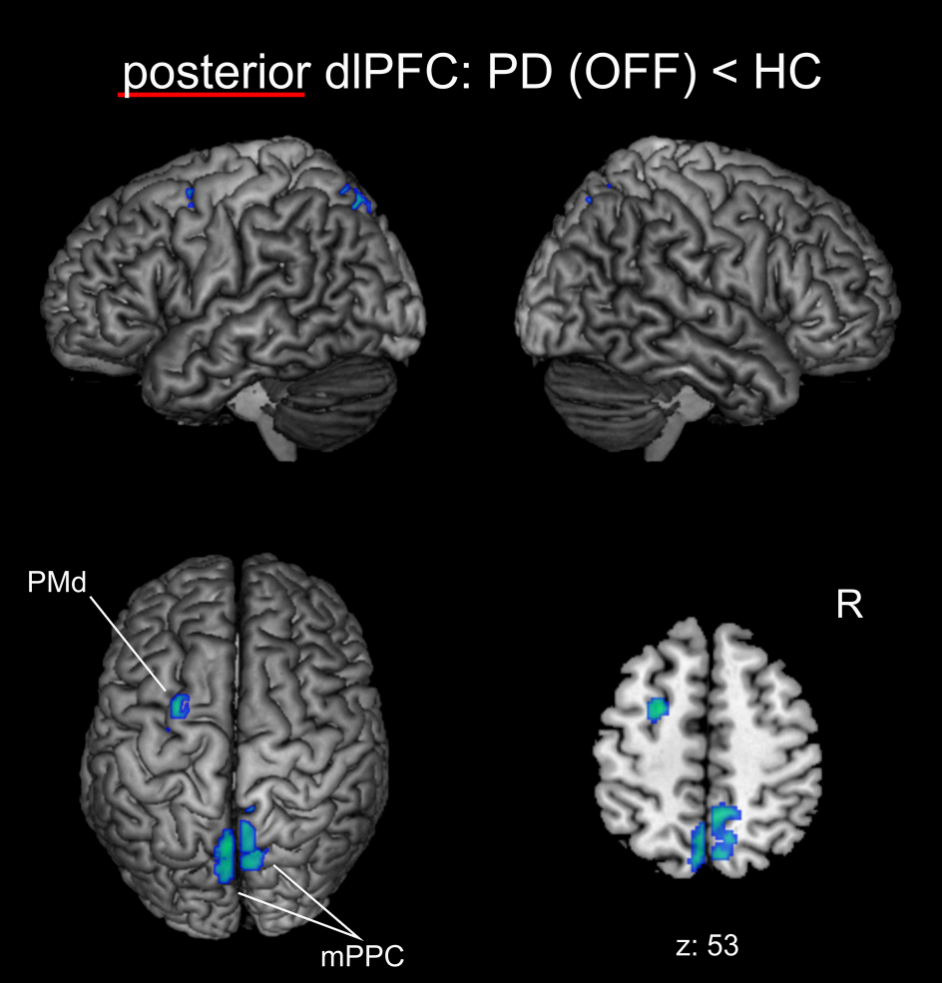
Functional connectivity changes between PD patients in the medical OFF state and healthy controls with the posterior right dlPFC seed projected onto the MNI single subject brain. Cold colors indicate regions with significantly decreased functional connectivity. Left lateral, right lateral, and top views as well as a representative axial section are shown. Label under the axial section represents z-coordinate in MNI space. R: right side.

We found significantly reduced FC for the posterior right dlPFC seed in PD patients in the medical OFF condition compared to healthy controls with bilateral posterior parietal and left premotor regions (Figure 2, Table 3). These regions particularly comprised the bilateral medial posterior parietal cortex (mPPC), that is, the bilateral precuneus (medial aspect of area 7), partially overlapping with areas 7A and 7P (Scheperjans et al., 2008) of the superior parietal lobule on the left side and partially reaching into areas 5m, 5Ci, and 7A (Scheperjans et al., 2008) of the superior parietal lobule on the right hemisphere. Furthermore, there was decreased FC with the left dorsal premotor cortex (PMd) within the posterior parts of the superior frontal sulcus (area 6). There were no regions showing significantly decreased FC with the anterior dlPFC seed. We did not find any significant FC increases with either seed in PD patients OFF medication compared to controls.

**Table 3:** Regions with significant functional connectivity group difference.

**Comparison**	**Brain region**	**Overlap with cytoarchitectonic regions**	**MNI**			**Voxels**
			***x***	***y***	***z***	
**GROUP DIFFERENCES**						
p dlPFC: HC > PD (OFF)	mPPC R	5m, 5Ci, 7A	9	−56	51	406
	mPPC L	7A, 7P	−4	−67	55	140
	PMd L		−25	1	52	122
p dlPFC: HC > PD (ON)	mPPC R	5m, 5Ci	10	−50	49	169
	mPPC L	5m, 5Ci, 7A	−7	−47	50	113
p dlPFC: PD (ON) > HC	dmPFC R		15	38	44	211
	dmPFC L		−15	48	34	69
Interaction: p dlPFC > a dlPFC × HC > PD (OFF)	mPPC L	7A, 7P	−8	−65	56	140
Interaction: a dlPFC > p dlPFC × HC > PD (OFF)	Lateral posterior cerebellum L	Lobule VIIa: Crus I + Crus II	−32	−76	−34	128

*p dlPFC, posterior right dlPFC seed; a dlPFC, anterior right dlPFC seed; HC, healthy control; PD (OFF), Parkinson patient in medical OFF condition; PD (ON), Parkinson patient in medical ON condition; R, right hemisphere; L, left hemisphere*.

**Figure 3:**
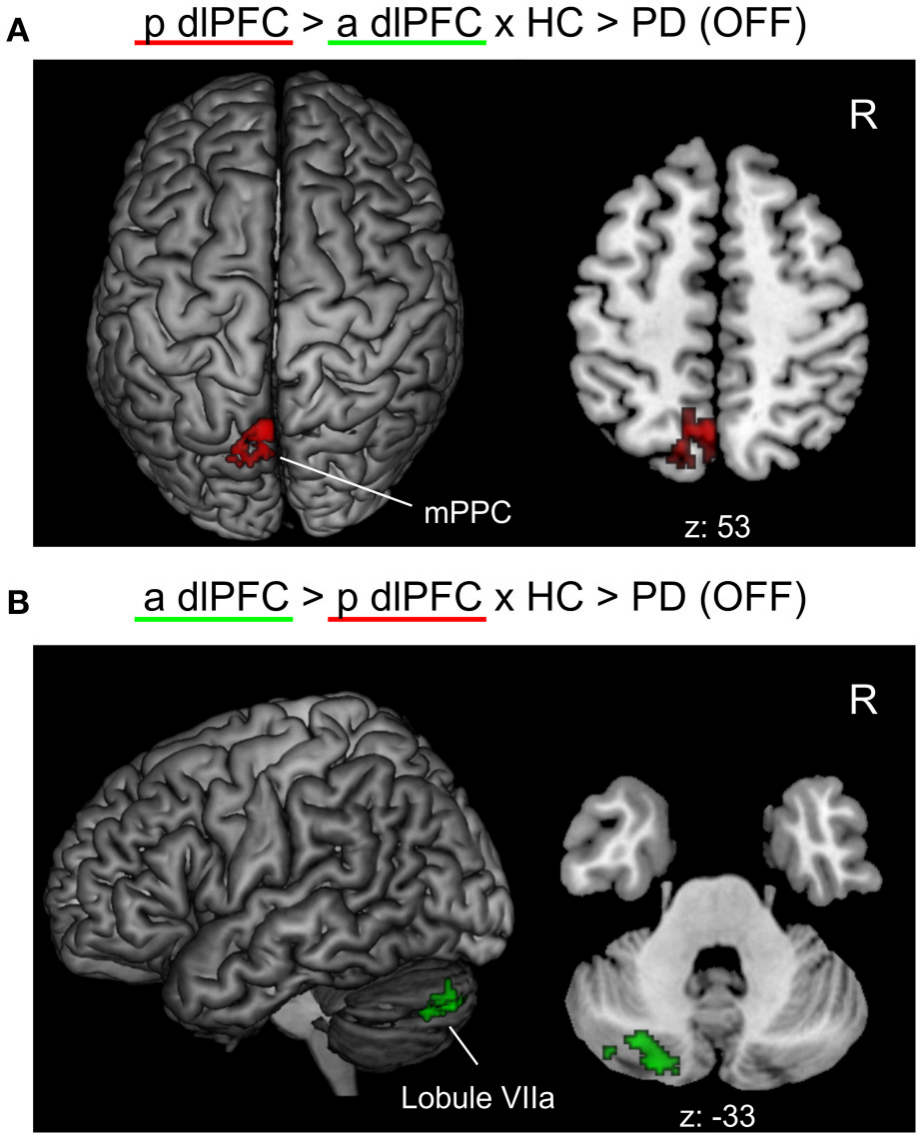
Significant “seed × subject group” interaction effects of the functional connectivity analysis between PD patients in the medical OFF and healthy controls projected onto the MNI single subject brain. “p dlPFC”: posterior right dlPFC seed; “a dlPFC”: anterior right dlPFC seed. **(A)** Interaction in the direction of a PD-related FC decrease of the posterior right dlPFC seed. Top view and a representative axial section are shown. **(B)** Interaction in the direction of a PD-related FC decrease of the anterior right dlPFC. Left lateral view and a representative axial section are shown. Labels under axial sections represent z-coordinates in MNI space. R: right side.

When testing for specific connectivity differences, i.e., the “seed × subject group” interaction (in the direction of a PD-related posterior right dlPFC connectivity decrease) for the medical OFF condition (Figure 3), we found a significant effect in the left mPPC, i.e., within the precuneus and adjacent superior parietal lobule overlapping with areas 7A and 7P. This indicates that the PD-related FC decrease with this region was specific to the posterior compared to the anterior right dlPFC seed. Furthermore, there was an interaction (in the direction of a PD-related anterior right dlPFC connectivity decrease) in Crus I and Crus II of lobule VIIa in the left posterior cerebellar lobe (Diedrichsen et al., 2009).

**Figure 4:**
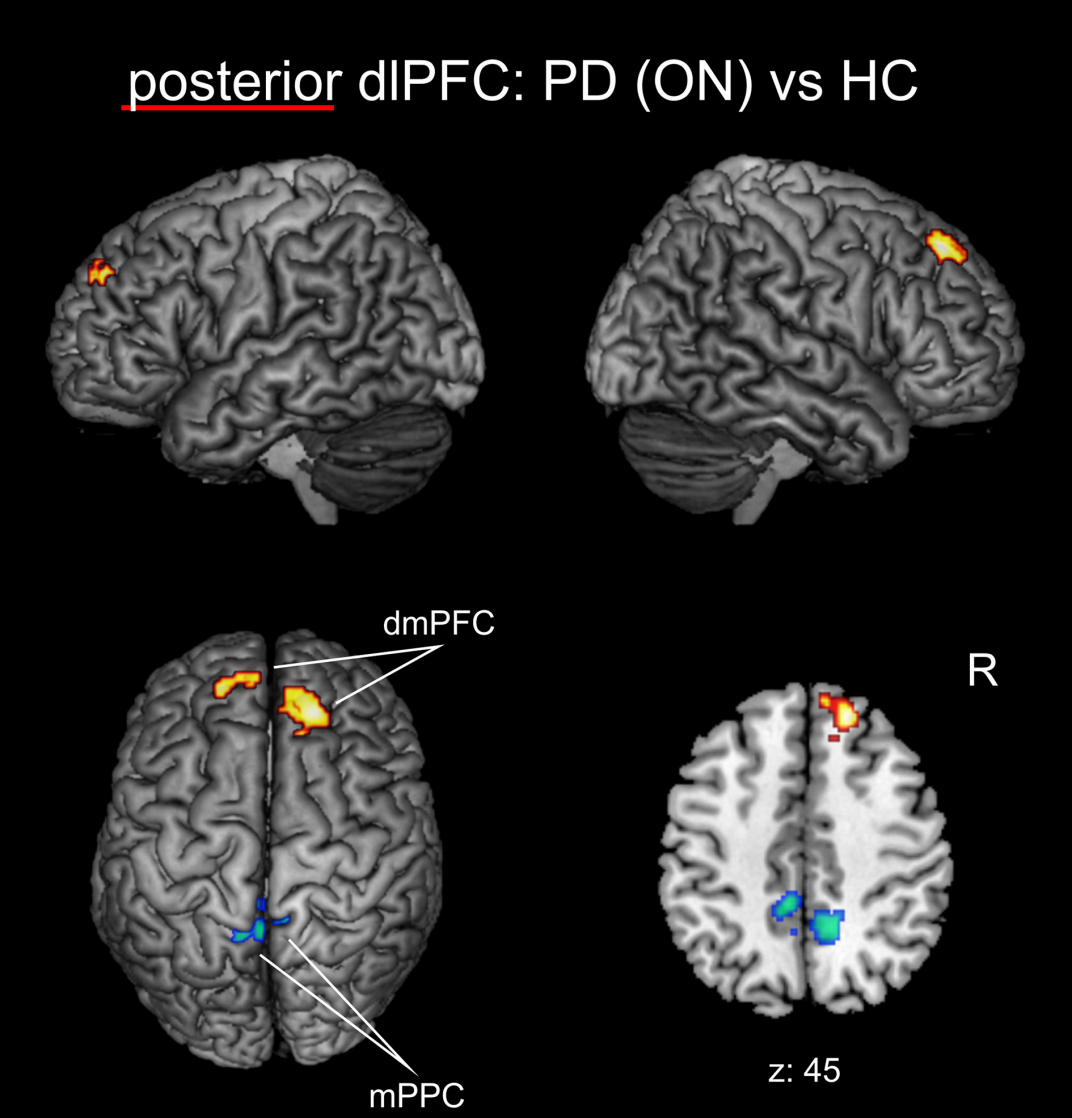
Functional connectivity changes between PD patients in the medical ON state and healthy controls with the posterior right dlPFC seed projected onto the MNI single subject brain. Hot colors indicate regions with significantly increased, cold colors regions with significantly decreased functional connectivity. Left lateral, right lateral, and top views as well as a representative axial section are shown. Label under the axial section represents z-coordinate in MNI space. R: right side.

When investigating the medical ON condition of PD patients compared to healthy controls (Figure 4, Table 3), there was a significant decrease of FC between posterior right dlPFC and the bilateral mPPC, i.e., the precuneus, partially reaching into areas 5m, 5Ci of the superior parietal lobule in both hemispheres and additionally into area 7A of the left hemisphere. Furthermore, we found a significant increase of FC between posterior right dlPFC and the dorsomedial aspects of the superior frontal gyrus bilaterally, corresponding to the dorsomedial prefrontal cortex (dmPFC, area 9). There were no significant FC alterations for the anterior right dlPFC seed between PD patients in the medical ON state and healthy controls.

For both seeds, there were no significant “seed × subject group” interactions for the group comparison between PD patients in the medical ON condition and healthy controls in either direction.

### Medication effect: PD medical ON vs. PD medical OFF

We found no significant FC alterations between the medical ON and medical OFF state of PD patients for either seed in the respective whole-brain FC GLM (*p* < 0.05, cluster-level FWE corrected).

When testing for medication effects in the *post-hoc* network analysis, using the extracted VOIs of regions with significantly altered FC of the right dlPFC seeds between PD patients and HC from the whole-brain group comparisons, there was a significant increase of FC for posterior right dlPFC with left and right dmPFC (each *p* < 0.05) in the medical ON compared to the medical OFF state of PD patients. There were no significant medication effects for posterior right dlPFC FC with left or right mPPC or for left PMd.

### Correlations with clinical parameters

We did not observe any significant whole-brain FC correlation with disease duration, UPDRS-III, MDRS, MoCA, or LED within the positively correlated networks of the seeds in either medical condition.

When specifically testing for correlations with FC of altered connections, we identified a significant negative correlation between disease duration and FC of the posterior right dlPFC seed with right mPPC in the medical OFF condition (*r* = −0.39; *p* < 0.05). There were no further significant correlations with disease duration in the medical ON or OFF state.

There were no significant correlations with UPDRS-III in either medical state and there were no correlations of the altered connections with MDRS or MoCA. We found no correlations with LED, neither for FC in the medical ON, nor for the medical ON vs. medical OFF FC difference. There was no significant correlation of disease duration with FC difference between ON and OFF.

## Discussion

We have investigated the FC of two subregions within the right dlPFC that are located at the inferior frontal sulcus and have been shown to be particularly involved in cognitive action control (Cieslik et al., 2013). We revealed considerable FC changes for the posterior subdivision of the right dlPFC in PD patients, while its anterior part showed no significant disease-related alterations. In particular, there was decreased FC of the posterior right dlPFC seed with the mPPC bilaterally as well as with left PMd. FC between posterior right dlPFC and right mPPC negatively correlated with disease duration, indicating that FC between these regions progressively decreases with advancing disease. While the FC decrease in the PMd was largely restored by dopaminergic treatment, the disruption of FC with bilateral mPPC persisted in the medical ON condition of PD patients. Furthermore, we identified an increase of FC induced by dopaminergic medication between the posterior right dlPFC seed and the bilateral dmPFC.

In the following sections, we will discuss our results in comparison to previous neuroimaging studies in PD, in the context of the functional role of right dlPFC and the hierarchical distinction of its subregions, and by providing functional interpretations of the identified regions showing altered connectivity.

### Comparison to previous neuroimaging studies

To our knowledge, this is the first report specifically investigating the FC of the right dlPFC in PD. Neuroimaging studies in PD have repeatedly shown a pathologic involvement of the dlPFC in association with disease-related impairment of EF. For example, a correlation of EF impairment in PD with gray-matter atrophy of the right dlPFC was revealed by voxel-based morphometry (Nagano-Saito et al., 2005).

Furthermore, a decreased activation of the dlPFC related to self-initiated movements in PD patients compared to healthy controls was revealed by regional cerebral blood flow measurements using positron emission tomography (PET) (Jahanshahi et al., 1995) and by fMRI (Disbrow et al., 2013). Another fMRI study using a working memory paradigm showed decreased activity in the dlPFC in PD patients showing impairment in EF compared to cognitively unimpaired PD patients and healthy controls (Lewis et al., 2003). In contrast, several other fMRI studies showed a PD-related increase of dlPFC activity, e.g., using a set-shifting paradigm (Monchi et al., 2004, 2007) or a Tower-of-London planning and a spatial working memory task (Cools et al., 2002). In the latter study, the task-related right dlPFC hyperactivation could be alleviated by dopaminergic medication.

Similarly, Nakamura et al. (2001) found an increased activation in right dlPFC, left PMd, and bilateral precuneus related to retrieval of a motor-sequence task using H_2_O^15^ PET. Comparable results showing increased activity of these regions related to sequence learning could also be revealed by another PET study (Mentis et al., 2003). The PD-related hyperactivation of the mentioned regions has already been discussed as a compensation for a disease induced disruption of that “retrieval network” in PD (Nakamura et al., 2001). This interpretation resonates well with our current results, which demonstrate a PD-related connectivity decrease within the very same regions of this network, i.e., between the right dlPFC, the left PMd, and the precuneus.

Indeed, all stereotaxic coordinates provided by the aforementioned studies fit well to the location of the posterior seed used in the current analysis and are clearly located posteriorly to the anterior seed. This is well in line with our finding that only the posterior right dlPFC shows FC alterations in PD and the anterior does not.

Beside these task-based functional neuroimaging studies, there are also some reports, where the dlPFC was revealed as a region with altered functional connectivity in PD. However, these alterations were mainly revealed for the connectivity between the dlPFC and the basal ganglia. For example, Wu et al. (Wu et al., 2012) found a decreased FC between the substantia nigra pars compacta and the dlPFC, which was partially alleviated by dopaminergic treatment. Furthermore, reduced FC between the basal ganglia network and the dlPFC was found in an independent component analysis (ICA) approach on resting-state fMRI data in PD (Szewczyk-Krolikowski et al., 2014). The concurrent decreased connectivity of the precuneus with this network is in line with our current findings. Another ICA study found decreased coupling of the right dlPFC to the fronto-parietal control network in mild-cognitively impaired PD patients (Amboni et al., 2015). However, the reported region of altered connectivity within the prefrontal cortex is far more posterior than our seed regions. Similarly to previous task-based fMRI studies, Trujillo et al. (2015) found an increased task-related activation of the bilateral dlPFC in a visuospatial working memory task. In contrast to our current findings, they revealed decreased FC between the dlPFC and the precuneus only for the left dlPFC and not for its right side. However, the sample size in this study was rather low and only newly diagnosed and untreated (“*de novo*”) PD patients were included.

Hence, whereas our findings are well in line with most task-based neuroimaging studies focusing on working-memory and sequence learning, the sparse mentioning of dlPFC FC alterations is only partly in line with the present results. However, this is the first study specifically investigating the whole-brain functional connectivity of the dlPFC in a large patient sample and all aforementioned FC studies were differentially focused, used quite different analysis approaches or seed regions, and comprised far less included patients. Our results can be explained well in accordance with current concepts of prefrontal function and its impairment in PD, which will be done in the following sections.

### Distinct connectivity changes for the anterior and posterior right dlPFC seeds in relation to executive function

The dlPFC is a key structure underlying EF in the human and non-human primate brain (Miller and Cohen, 2001; Hoshi, 2006). It accomplishes its functions in “top-down” control of behavior by monitoring ongoing actions and the external environment, collating them with internal goals and states, and consecutively adjusting actions and behavior (Ridderinkhof et al., 2004a; Funahashi and Andreau, 2013). In this regard, the dlPFC is particularly involved in integrating information from all sensory systems and manipulating working-memory information and action-plans for goal-directed behavior (Goldman-Rakic, 1995; Miller and Cohen, 2001; Petrides, 2005). This is facilitated by an extensive interconnection of the dlPFC with associative parietal and temporal areas (Cavada and Goldman-Rakic, 1989; Petrides and Pandya, 1999), premotor regions (Bates and Goldman-Rakic, 1993; Lu et al., 1994), subcortical structures (Alexander et al., 1986), as well as with other prefrontal regions (Barbas and Pandya, 1989).

In PD, alterations in dlPFC function are mainly attributed to a diminished effectiveness of frontostriatal projections to the dorsal aspects of the caudate head due to dopaminergic loss in the nigrostriatal pathway, and to a lesser extend also to dopamine depletion in the prefrontal cortex itself via the mesocortical pathway (Owen, 2004; Leh et al., 2010).

The current results indicate that FC of the right dlPFC is differentially affected with respect to subdivisions of this region, as only the posterior aspect of the right dlPFC seed shows FC alterations in PD, while the anterior part does not. Recent research of the lateral prefrontal cortex in healthy subjects has highlighted a posterior-anterior directed functional and hierarchical gradient of the prefrontal cortex and the dlPFC in particular (Koechlin et al., 2003; Badre and D'Esposito, 2009; Taren et al., 2011). While the posterior parts of the dlPFC facilitate rather basal aspects of EF by means of sensory input integration and simple stimulus-to-response mappings, increasingly abstract rule-based processing for goal-directed behavior emerges in the anterior parts of the dlPFC. This hierarchical gradient is also reflected at the connectional level, as the posterior dlPFC shows diverse connections to parietal associative regions for stimulus integration and selection, while the anterior dlPFC is more strongly connected to other regions within the prefrontal cortex for its function in top-down action control and manipulation of working memory (Petrides and Pandya, 1999, 2007; Badre and D'Esposito, 2009). The functional characterization and FC of the two right dlPFC seed-regions revealed by our previous co-activation based parcellation study (Cieslik et al., 2013), are well in line with this hierarchical model of the dlPFC. The study revealed that the posterior right dlPFC cluster was more involved in rather basal aspects of EF like stimulus integration and working memory, while the anterior right dlPFC seed had more abstract, supervisory function being involved in conflict resolution and more complex EF tasks like the Stroop task or go/no-go trials.

As only the posterior right dlPFC seed showed considerable FC alterations in PD in the current analysis, it can be inferred that the posterior dlPFC circuits for rather basal EF like stimulus integration and working memory updating are more severely affected in PD than the connections of the anterior dlPFC cluster, which is involved in more abstract functions of EF like action inhibition and set-shifting.

Even though there are reports showing that working memory updating is more strongly affected in early PD than set-shifting abilities (Ranchet et al., 2011), higher EF like set-shifting and inhibition are typically also found to be impaired in PD patients (Kensinger et al., 2003; Kudlicka et al., 2011; Dirnberger and Jahanshahi, 2013). An obvious explanation for these findings could be seen in a hierarchical dependency in dlPFC processing, where the higher EF build on the processing of the basal dlPFC levels. In this sense, the PD-related impairment of posterior right dlPFC leads to dysfunction for the basal as well as for the abstract aspects of dlPFC function through bottom-up processing. However, another possible explanation might be a differing involvement of both dlPFC subdivisions in dissociable dopamine-dependent functional systems. It has been shown that cognitive flexibility in PD, that is set-shifting ability and inhibition, is mainly dependent on dopamine levels in the striatum and activity of the subthalamic nucleus (Cools, 2006; Monchi et al., 2007; Jahanshahi et al., 2015). In contrast, cognitive stability, in terms of working memory retrieval and maintenance, is stronger dependent on dopamine levels in the prefrontal cortex and less on striatal function (Cools et al., 2002; Cools, 2006; Monchi et al., 2007). Hence, deficits in higher EF might be mainly driven by pathologic signaling in the basal ganglia loop through dopaminergic depletion in the nigrostriatal pathway leading to a consecutive secondary decrease of activation in the dlPFC through frontostriatal projections (Monchi et al., 2007). In this setting, coupling between the anterior dlPFC seed and the striatum as well as its connections to other cortical regions may possibly be unaffected. In contrast, the deficits of lower EF might be more strongly driven by dopaminergic loss of prefrontal neurons via the mesocortical pathway and consecutive cortico-cortical imbalances (Martinu et al., 2012), which may be reflected by the FC disturbances of the posterior dlPFC seen in our data.

### Functional relevance of altered connections with right posterior dlPFC

In the current analysis, the bilateral mPPC was the main region displaying disrupted connectivity with the posterior right dlPFC cluster. Correlation analysis additionally revealed a negative correlation between disease duration and FC between the posterior seed and right mPPC indicating that this region is progressively decoupled from posterior right dlPFC with advancing disease. It is known from tracing studies in non-human primates that the mPPC is anatomically connected to the dlPFC (Cavada and Goldman-Rakic, 1989; Petrides and Pandya, 1999; Leichnetz, 2001), which is also reflected by their task-based and task-independent FC (Margulies et al., 2009; Bzdok et al., 2015). Besides other functions, the mPPC is associated with the allocation of spatial attention and control of visually/spatially guided actions (Cavanna and Trimble, 2006; Bzdok et al., 2015). In particular, co-activation of the right dlPFC and mPPC has been linked to monitoring and manipulation of visuo-spatial working-memory in a mental rotation task (Suchan et al., 2002). In this context, the involvement of dlPFC has been attributed to active and manipulative processing of working memory compared to more passive processing in the ventrolateral prefrontal cortex (Suchan, 2008). However, another fMRI study proposed that the dlPFC is more strongly involved in the monitoring of working-memory information, while manipulation is performed in the posterior parietal cortex (Champod and Petrides, 2007). This is in line with the theory of Petrides (2005) on the organization of the prefrontal cortex, who postulated an involvement of the dlPFC in stimulus manipulation secondary to its primary role in monitoring. Given this evidence, the PD-related decoupling between the posterior right dlPFC seed and mPPC demonstrated by our analysis might thus point to impairment in the interaction between both regions for visuo-spatial working-memory monitoring and manipulation. Behavioral studies indeed show that, even from the early stages of the disease on, PD patients reveal severe deficits in visuo-spatial working memory, which can be ameliorated by dopaminergic treatment (Costa et al., 2003; Mollion et al., 2003). However, the impaired interaction between dlPFC and mPPC in our study seems not to be directly alleviated by dopaminergic treatment, as the FC decrease between these regions remained largely unaffected in the medical ON compared to the medical OFF state. A possible explanation for this finding might be that the deficits in visuo-spatial working memory processing caused by decoupling of these regions are compensated through dopamine-induced recruitment of other brain regions in the medical ON state.

A possible candidate for such compensatory area is the dmPFC. Additionally to the further diminished FC in the mPPC, the comparison between PD patients and healthy controls in the medical ON-state yielded an increased FC between the posterior right dlPFC seed and the bilateral dmPFC in medicated patients. The medial PFC is known to strongly interact with dorsolateral and ventrolateral prefrontal cortex in order to facilitate EF (Ridderinkhof et al., 2004a; Sallet et al., 2013). In this network of prefrontal regions, medial PFC has been implicated in performance monitoring of actions, action selection and conflict detection for goal-directed behavior (Ridderinkhof et al., 2004b; Desmet et al., 2011). In particular, current actions are evaluated in medial PFC in accordance with personal goals and expected rewards in order to initiate behavioral adjustments, e.g., by the dlPFC (Ridderinkhof et al., 2004b). It should be mentioned that the monitoring function of the medial PFC was mostly assigned to regions more ventrally than the dmPFC regions revealed in the current study, i.e., in the ACC (Matsumoto and Tanaka, 2004; Ridderinkhof et al., 2004a). Nevertheless, several fMRI studies showed that the dmPFC dorsally to the ACC is additionally active in monitoring, in particular under conditions, when more abstract rules have to be applied and complex decisions have to be made (Goel and Dolan, 2000; Volz et al., 2003; Desmet et al., 2011). In this regard, the dmPFC has also been reported in association with intentional action control (Brass and Haggard, 2008), that is decisions on whether an action should be performed or not, and with self-referential judgements (Northoff and Bermpohl, 2004).

Taken together, the increased connectivity between the posterior right dlPFC cluster and bilateral dmPFC induced by dopaminergic treatment in patients may probably be related to a dopamine-induced recruitment of additional action monitoring resources that can be regarded as a compensatory mechanism to restore EF. Particularly, the monitoring function of dmPFC for adjusting behavior might be intensified by dopaminergic medication in order to compensate for the PD-related deficits in working memory updating, monitoring and manipulation.

Interestingly, previous studies in healthy controls have shown that administration of dopaminergic drugs increased performance of action monitoring, while antidopaminergic medication diminished monitoring function (de Bruijn et al., 2004; Zirnheld et al., 2004). In these studies, the recorded alterations of event-related potentials (ERP) over medial prefrontal cortex in action monitoring tasks have again mainly been attributed to the ventrally located ACC, i.e., ventrally to the regions of significantly increased connectivity in our study. However, given the aforementioned evidence that both, the ACC and the dmPFC, are involved in conflict detection on different levels of abstraction (Desmet et al., 2011), it is conceivable that the midline ERP recordings are also, at least partly, induced by this dorsally adjacent region. Hence, the activity of the medial PFC seems to be altered by (anti-) dopaminergic treatment regarding its function for action monitoring and conflict detection, which is well in line with our current findings.

Medial PFC has also been associated with impulsivity disorders in PD in the context of dopaminergic stimulation, as being a target of an “overdose” effect of dopaminergic medication in PD (Voon et al., 2011; Antonelli et al., 2014). However, activations of the medial PFC related to impaired impulsive control are typically found rostrally to the dmPFC regions found in our study (e.g., Horn et al., 2003; Antonelli et al., 2014). Hence, we would rate a dopamine induced disturbance of impulsivity control as an explanation for the increased connectivity between bilateral dmPFC and the posterior right dlPFC seed in the medical ON state as less probable.

In contrast to the decoupling with mPPC, FC decrease of the right posterior dlPFC seed with left PMd was only revealed in the medical OFF condition. Thus, altered connectivity between posterior right dlPFC and left PMd seems to be recovered through dopaminergic medication.

It is known from monkey studies, that PMd is a major target of the structural efferents from the dlPFC (Barbas and Pandya, 1987; Petrides and Pandya, 1999; Luppino et al., 2003). It is thought, that PMd facilitates premotor sequencing of visuo-spatial information in its rostral parts and generation of motor plans more caudally. Via its functional connections, the dlPFC modulates these PMd functions by attentional selection of relevant information (Abe and Hanakawa, 2009). It has been shown that, while the right dlPFC shows decreased activation in PD, the PMd reveals a hyperactivation related to movement tasks in PD, which has mainly been attributed to a compensatory mechanism (Sabatini et al., 2000). Furthermore, a PD- related disconnection between right dlPFC and PMd in tasks related to action attention (Rowe et al., 2002), movement selection and initiation (Wu et al., 2011) has been reported, which is well in line with the decreased connectivity between the posterior right dlPFC seed and the left PMd in PD patients in the current study. This disconnection can, hence, be interpreted as a disruption of the modulating and controlling function of the dlPFC over premotor cortex function. This dysfunction seems to be alleviated by dopaminergic treatment, which resonates well with the notion that PMd function and its connection to primary motor cortex are restored after levodopa administration (Buhmann et al., 2004; Baumer et al., 2009).

Although, there were no significant group differences in whole-brain FC between PD patients and healthy controls for the anterior dlPFC cluster, we found a significant “seed × subject group” interaction in the direction “anterior > posterior right dlPFC seed × healthy controls > PD patients” in the left lateral posterior lobe of the cerebellum, i.e., in Crus I and Crus II of lobule VIIa. This can be interpreted as a seed specific PD-related FC decrease, i.e., that the FC between the anterior right dlPFC seed and the left lateral cerebellum shows a significantly stronger decrease in PD than between the posterior right dlPFC cluster and this cerebellar region. In PD, several alterations in the functional activation and connectivity of the cerebellum have been reported related to various motor and cognitive symptoms of the disease (see Martinu and Monchi, 2013; Wu and Hallett, 2013 for reviews). The cerebellum is reciprocally connected to the prefrontal cortex via the cerebello-thalamo-cortical circuit. In particular, the (anterior) dlPFC has projections to Crus I and Crus II of the cerebellar cortex, which again project back to the prefrontal cortex over the dentate nuclei and mediodorsal thalamus (Middleton and Strick, 2001; Kelly and Strick, 2003; Balsters et al., 2010). The connection between the prefrontal cortex and the lateral posterior cerebellar lobe including Crus I and Crus II has been primarily linked to be involved in the processing of cognitive tasks and EF, e.g., in working memory and complex decision-making (Bellebaum and Daum, 2007; Stoodley and Schmahmann, 2010). In this regard, the cerebral-cerebellar connections involved in these processes are typically contralateral, i.e., that the left cerebellar hemisphere is functionally connected with right-hemispheric cerebral regions (Stoodley and Schmahmann, 2010), which is well in line with the FC alteration between the right dlPFC cluster and left cerebellar lobule VIIa in the observed interaction of our study. Furthermore, the left lateral posterior lobe of the cerebellum has been stronger related to working memory, EF and spatial processing, while its right side was more involved in language tasks (Stoodley and Schmahmann, 2009). Since the anterior right dlPFC seed is associated with more complex and abstract processing for EF compared to the posterior seed, as outlined above, the observed interaction within left Crus I and Crus II might indicate, that, in PD, the prefrontal-cerebellar connections facilitating EF are more strongly affected for the higher-order cognitive processes associated with the anterior right dlPFC cluster than for the rather basal EF related to the posterior seed.

Although, the striatum and consecutive basal ganglia loop is the main projection of the dlPFC (Alexander et al., 1986; Cools et al., 2001) and there is ample evidence that processing in the frontostriatal circuit is impaired in PD (Owen, 2004; Monchi et al., 2007; Martinu et al., 2012; Nagano-Saito et al., 2014), we did not observe significant connectivity changes of the right dlPFC clusters with structures of the basal ganglia. Existing evidence on altered FC between prefrontal regions and the striatum is quite inconsistent, as there are single seed-to-target FC analyses showing FC alterations (Kwak et al., 2010), while two seed-to-whole-brain analyses of several striatal seeds also revealed no dorsolateral frontostriatal connectivity changes (Helmich et al., 2010; Hacker et al., 2012). Hence, the existence of these PD related dorsolateral frontostriatal connectivity alterations is doubtful, and dysfunction of the frontostriatal pathway may possibly result from a congruent deficit of the dlPFC and striatum through dopaminergic depletion along the nigrostriatal pathway (Owen, 2004), while leaving the connectivity between both regions unaffected. On the other hand, it is conceivable, that possible effects in the basal ganglia are missed due to their small sizes in the current seed-to-whole-brain approach, as effects of small structures are easily obliterated by the cluster-level thresholds for multiple-comparison correction.

### Limitations

We have to admit several limitations in our study. First, the included sample of PD patients is quite heterogeneous regarding disease stages and disease duration, as we did not restrict study inclusion to specific disease states or functional criteria. However, we would argue that this heterogeneity increases variability across the disease-related connectivity alterations of the several disease stages, which increases the generalizability and robustness of the observed results.

Our data does not comprise specific EF test scores to directly substantiate our hypotheses on EF impairment through dlPFC connectivity changes via correlation analyses. The cognitive scores available in our data are the MoCA and MDRS score. MoCA is a global cognitive test battery, which incorporates several tests addressing EF, but does not exclusively focus on EF. The MDRS mainly targets to evaluate (mild) cognitive impairment and dementia, while its tested cognitive spectrum only partly taps EF. Since there is strong evidence for the association of dlPFC function and its connectivity to EF from the literature, as outlined in the discussion, the inferences on EF impairment drawn in our study appear very likely. Nevertheless, these inferences need to be validated by future studies, which incorporate specific EF testing to proof the associations between PD related FC changes of the dlPFC and EF impairment. Our study conducted on a very large clinical sample of PD patients and enhanced by measurements in medical ON and OFF states yields substantial evidence on the connectivity changes of the right dlPFC in PD and, thus, provides a strong basis to implement such studies with a specialized functional focus or on specific patient subsamples.

Medication protocols across patients were not uniform, as each PD patient received a specific dopaminergic medication scheme resulting from an individual optimization of treatment. Hence, there was a rather high diversity of possible combinations of medication. From our point of view, this diversity in treatment regimens increases variability within the patient group and should make the observability of significant effects between patients and controls or between the medication ON and OFF states of patients less probable, rendering our results more independent from single drug-specific effects. To make antiparkinsonian treatment plans of the included PD patients more comparable and express the dose intensity consistently on a single scale, we calculated the levodopa equivalent dose (LED) and correlated it with observed FC. Here, we did not find a significant effect of LED on FC of either right dlPFC seed.

Our analysis focused on PD-related FC alterations of a functionally well-defined region within the right dlPFC. Although, bilateral dlPFC plays a role in EF in general, evidence shows that cognitive action control is strongly lateralized to the right dlPFC (e.g., Aron et al., 2004; Nee et al., 2007; Vogt et al., 2007). This observation could be objectified by a BrainMap based meta-analysis indicating that 76% of neuroimaging experiments involving “action” and “cognition” activated the right dlPFC VOI used in our study but not its left-hemispheric counterpart (Cieslik et al., 2013). The right dlPFC region underlying the current analysis was derived from the conjunction of 4 fMRI experiments investigating cognitive motor control (Jakobs et al., 2009; Cieslik et al., 2010; Eickhoff et al., 2011; Kellermann et al., 2012), where congruent activation across these studies was exclusively found within this right dlPFC region. This region could then be subdivided into the anterior and posterior subregion by co-activation based parcellation (Cieslik et al., 2013). As the seed definition and parcellation cannot implicitly be transferred to the left hemisphere and due to the aforementioned evidence for the strong lateralization of the dlPFC for cognitive action control, we focused our current analysis on the well-defined right anterior and posterior dlPFC seed regions.

### Conclusion

Our analysis revealed that, from the two seed regions within the right dlPFC involved in cognitive action control, only the posterior subdivision features distinct FC changes in PD. The observed changes relate to PD-specific impairments in EF in terms of impaired stimulus integration, working memory updating and cognitive stability, which are mainly attributed to the posterior dlPFC. There is a PD-related decoupling of the posterior right dlPFC cluster from the bilateral mPPC, which points to a disruption of visuo-spatial integration for EF. Interestingly, this loss of connectivity was not directly alleviated by dopaminergic treatment. The increased coupling between the posterior right dlPFC seed and the bilateral dmPFC in medically treated patients might indicate a dopamine-related increase in action monitoring and adjustment to compensate for EF deficits. Our findings build a strong basis for the understanding of prefrontal cortex connectivity changes in PD. However, the related functional inferences on EF impairment need further validation by future studies incorporating specific EF testing.

## Author contributions

SE, JC, FH, MS, and CM substantially contributed to the conception and design of the work. All authors substantially contributed to the analysis and interpretation of data for the work. JC and SE drafted the work. All authors revised the work critically for important intellectual content. All authors finally approved the manuscript version to be published. All authors agree to be accountable for all aspects of the work in ensuring that questions related to the accuracy or integrity of any part of the work are appropriately investigated and resolved.

## Ethics statement

The study has been performed in accordance with the ethical standards of the Declaration of Helsinki and was approved by the local ethics committee of the Heinrich-Heine-University Düsseldorf Medical Faculty. All subjects provided written informed consent to participate in the study prior to inclusion.

### Conflict of interest statement

The authors declare that the research was conducted in the absence of any commercial or financial relationships that could be construed as a potential conflict of interest. The reviewer LH and handling Editor declared their shared affiliation, and the handling Editor states that the process nevertheless met the standards of a fair and objective review.

## Abbreviations

ACC: anterior cingulate cortex
ALE: activation likelihood estimation
dlPFC: dorsolateral prefrontal cortex
dmPFC: dorsomedial prefrontal cortex
DVARS: root mean squared signal change across time-series
EF: executive function
ERP: event-related potentials
FC: functional connectivity
FD: framewise displacement
FWE: family wise error
GLM: general linear model
HC: healthy controls
ICA: independent component analysis
MDRS: Mattis dementia rating scale
MoCA (score): Montreal cognitive assessment (score)
mPPC: medial posterior parietal cortex
LED: levodopa equivalent dose
PET: positron emission
PD: Parkinson's disease
PMd: dorsal premotor region
RMS: root mean squared
UPDRS-III: part III of the unified Parkinson's disease rating scale
VOI: volume of interest.
