# Supplementary material for: Differential Functional Connectivity Alterations of Two Subdivisions within the Right dlPFC in Parkinson's Disease
Source: Front Hum Neurosci. 2017 May 30;11:288. doi: 10.3389/fnhum.2017.00288 (PMC5447710; doi:10.3389/fnhum.2017.00288)
Supplement: Supplementary file 1 [file Table1.docx]

**Supplementary Table S1:** PD-related characteristics and measures of each patient in the sample. UPDRS-III: Unified Parkinson’s Disease Rating Scale part III; MDRS: Mattis Dementia Rating Scale. MoCA: Montreal Cognitive Assesment (MoCA was not available (n.a.) in 6 of 39 patients); LED: Levodopa equivalent dose; Motor subtypes: AR = akinetic-rigid, TD = tremor-dominant, MT = mixed type; Symptom lateralization: R = right dominant, L = left dominant.

| Subj. Nr | Disease duration | Hoehn & Yahr | UPDRS-III (medical OFF) | UPDRS-III (medical ON) | MDRS (medical ON) | MoCA (medical ON) | LED [mg] | Motor subtype | Symptom lateralization |
| --- | --- | --- | --- | --- | --- | --- | --- | --- | --- |
| 1 | 8 | 2 | 22 | 10 | 135 | 23 | 1474 | MT | R |
| 2 | 0 | 1.5 | 15 | 11 | 137 | 27 | 790 | MT | L |
| 3 | 6 | 3 | 34 | 20 | 142 | 27 | 1297 | MT | R |
| 4 | 9 | 3.5 | 45 | 40 | 142 | 28 | 1485 | MT | L |
| 5 | 13 | 3 | 37 | 19 | 142 | 27 | 998 | MT | R |
| 6 | 14 | 3.5 | 55 | 38 | 134 | 21 | 1325 | AR | R |
| 7 | 9 | 2 | 15 | 6 | 143 | 28 | 651 | AR | L |
| 8 | 12 | 2 | 33 | 26 | 135 | 19 | 930 | TD | R |
| 9 | 11 | 3 | 44 | 9 | 125 | n.a. | 366 | MT | L |
| 10 | 1 | 2 | 18 | 16 | 138 | n.a. | 640 | TD | L |
| 11 | 9 | 3.5 | 49 | 26 | 135 | 23 | 625 | AR | L |
| 12 | 18 | 3.5 | 27 | 12 | 143 | 28 | 1222 | MT | L |
| 13 | 6 | 3.5 | 53 | 28 | 143 | 28 | 737 | MT | L |
| 14 | 12 | 3 | 26 | 9 | 139 | 26 | 565 | MT | L |
| 15 | 2 | 2.5 | 23 | 13 | 141 | 28 | 875 | MT | L |
| 16 | 16 | 4 | 36 | 20 | 120 | n.a. | 675 | MT | R |
| 17 | 1 | 2 | 38 | 17 | 138 | 25 | 923 | MT | L |
| 18 | 9 | 3 | 36 | 15 | 139 | 21 | 1405 | MT | R |
| 19 | 17 | 3 | 55 | 26 | 137 | 22 | 1995 | AR | L |
| 20 | 1 | 2 | 18 | 14 | 143 | 29 | 100 | TD | L |
| 21 | 9 | 2 | 29 | 25 | 140 | 26 | 1397 | MT | L |
| 22 | 18 | 2.5 | 34 | 18 | 126 | 20 | 1011 | MT | L |
| 23 | 12 | 2.5 | 26 | 11 | 143 | 29 | 1573 | AR | R |
| 24 | 5 | 3 | 41 | 18 | 127 | n.a. | 1564 | MT | L |
| 25 | 21 | 3 | 39 | 15 | 137 | 17 | 1380 | MT | L |
| 26 | 7 | 3.5 | 38 | 21 | 144 | 25 | 679 | AR | L |
| 27 | 4 | 2.5 | 57 | 47 | 135 | 24 | 472 | MT | R |
| 28 | 7 | 2 | 22 | 14 | 142 | 28 | 950 | MT | R |
| 29 | 3 | 2 | 27 | 22 | 144 | 27 | 765 | MT | R |
| 30 | 20 | 2.5 | 37 | 15 | 136 | 19 | 1682 | AR | L |
| 31 | 3 | 2 | 34 | 28 | 132 | 20 | 1150 | AR | L |
| 32 | 14 | 3 | 38 | 14 | 137 | 24 | 1625 | AR | R |
| 33 | 8 | 3 | 42 | 9 | 137 | 23 | 1520 | AR | L |
| 34 | 1 | 1 | 20 | 15 | 143 | n.a. | 400 | MT | L |
| 35 | 6 | 2 | 31 | 19 | 142 | 29 | 1349 | TD | R |
| 36 | 11 | 4 | 40 | 40 | 118 | n.a. | 937 | AR | R |
| 37 | 3 | 2 | 51 | 46 | 137 | 16 | 465 | AR | R |
| 38 | 11 | 3 | 49 | 43 | 141 | 27 | 998 | AR | L |
| 39 | 10 | 2 | 38 | 29 | 136 | 24 | 905 | TD | R |
